# Prognostic value of ^18^F-FDG PET and PET/CT for assessment of treatment response to neoadjuvant chemotherapy in breast cancer: a systematic review and meta-analysis

**DOI:** 10.1186/s13058-020-01350-2

**Published:** 2020-10-31

**Authors:** Sangwon Han, Joon Young Choi

**Affiliations:** 1 Department of Nuclear Medicine, Asan Medical Centre, University of Ulsan College of Medicine, 88 Olympic-ro 43-gil, Songpa-gu, Seoul, 05505 Republic of Korea; 2 Department of Nuclear Medicine, Samsung Medical Centre, Sungkyunkwan University School of Medicine, 81 Irwon-ro, Gangnam-gu, Seoul, 06351 Republic of Korea

**Keywords:** Breast neoplasms, Fluorodeoxyglucose F18, Positron emission tomography, Neoadjuvant therapy, Prognosis

## Abstract

**Background:**

We performed a systematic review and meta-analysis to evaluate the prognostic significance of ^18^F-FDG PET and PET/CT for evaluation of responses to neoadjuvant chemotherapy (NAC) in breast cancer patients.

**Methods:**

We searched PubMed, Embase, and the Cochrane Library databases until June 2020 to identify studies that assessed the prognostic value of ^18^F-FDG PET scans during or after NAC with regard to overall (OS) and disease-free survival (DFS). Hazard ratios (HRs) and their 95% confidence intervals (CIs) were pooled meta-analytically using a random-effects model.

**Results:**

Twenty-one studies consisting of 1630 patients were included in the qualitative synthesis. Twelve studies investigated the use of PET scans for interim response evaluation (during NAC) and 10 studies assessed post-treatment PET evaluation (after NAC). The most widely evaluated parameter distinguishing metabolic responders from poor responders on interim or post-treatment PET scans was %ΔSUVmax, defined as the percent reduction of SUVmax compared to baseline PET, followed by SUVmax and complete metabolic response (CMR). For the 17 studies included in the meta-analysis, the pooled HR of metabolic responses on DFS was 0.21 (95% confidence interval [CI], 0.14–0.32) for interim PET scans and 0.31 (95% CI, 0.21–0.46) for post-treatment PET scans. Regarding the influence of metabolic responses on OS, the pooled HRs for interim and post-treatment PET scans were 0.20 (95% CI, 0.09–0.44) and 0.26 (95% CI, 0.14–0.51), respectively.

**Conclusions:**

The currently available literature suggests that the use of ^18^F-FDG PET or PET/CT for evaluation of response to NAC provides significant predictive value for disease recurrence and survival in breast cancer patients and might allow risk stratification and guide rational management.

## Introduction

Neoadjuvant chemotherapy (NAC) is the initial therapy for patients with inoperable or locally advanced breast cancer [1] and enables more patients with operable but large primary tumours to be treated with breast-conserving surgery [2]. Given a non-negligible proportion of patients treated with NAC cannot achieve an optimal response or have subsequent disease progression, accurate assessment of the therapeutic response is important to reduce toxicity from ineffective chemotherapy and guide selection of an alternative treatment option. Changes in tumour morphology and size on breast magnetic resonance imaging (MRI) after several cycles of NAC are widely used markers for assessment of the treatment response and prediction of patient outcome [3]. However, MRI studies have different predictive values across the various breast cancer subtypes [4, 5], and there is a limited evidence for their prognostic value. In addition, MRI techniques do not allow evaluation of newly developing distant metastasis during NAC.

Decreased glucose metabolism within breast cancer tissue on ^18^F-fluorodeoxyglucose positron emission tomography/computed tomography (^18^F-FDG PET/CT) is a useful indicator to assess the effectiveness of NAC [6]. Several meta-analyses have reported that ^18^F-FDG PET/CT scans performed during or after NAC could predict the final pathological response after completion of NAC [7–10]. A meta-analysis directly comparing PET/CT and breast MRI reported that PET/CT has a higher sensitivity and specificity for assessment of pathological response than conventional MRI when performed before 3 cycles of NAC [11]. More recently, accumulating evidence has suggested that assessment of the metabolic response using ^18^F-FDG PET or PET/CT has prognostic significance in breast cancer patients who underwent NAC [12–32].

The use of ^18^F-FDG PET or PET/CT for response evaluation is not yet established in clinical practice [1], in part due to lack of evidence supporting changes in treatment plans based on the results of PET scans performed to evaluate treatment responses and whether this strategy improves clinical outcomes [33]. However, prior to designing clinical trials evaluating the use of PET for response-adaptive treatment, a thorough review of the currently available data regarding the correlations between metabolic responses evaluated using PET or PET/CT scans and disease recurrence or survival, and of associated risk stratification of breast cancer patients during or after NAC, is warranted. In addition, differences in the timing of PET scans, response criteria and their threshold values across the available studies and their potential effects on survival also need to be assessed. Therefore, we performed a systematic review and meta-analysis of the currently available literature on the prognostic value of ^18^F-FDG PET or PET/CT for treatment response evaluation in breast cancer patients who underwent NAC.

## Materials and methods

This meta-analysis adhered to the Preferred Reporting Items for Systematic Reviews and Meta-Analyses (PRISMA) guidelines [34]. The protocol was registered in the International Prospective Register of Systematic Reviews (PROSPERO) network (registration no.CRD42020188979).

### Literature search and data extraction

The PubMed, Embase, and the Cochrane Library databases were searched from inception to June 4, 2020. Search queries included the related terms ‘breast cancer’, ^18^F-FDG PET’, ‘neoadjuvant therapy’, and ‘prognosis’, which are described in the Additional File 1. There was no language restriction for the electronic searches. The references of the extracted articles were also examined to identify additional relevant articles.

The inclusion criteria were based upon the Patient/Intervention/Comparator/Outcome/Study design (PICOS) criteria as follows [34]: (1) female ‘patients’ with breast cancer; (2) ^18^F-FDG PET, PET/CT, or PET/MRI during or after NAC as ‘intervention’; (3) no ‘comparator’ for the study; (4) overall (OS) and disease-free survival (DFS) as ‘outcome’; and (5) prospective or retrospective studies published as original articles as ‘study design’. The exclusion criteria were as follows: (1) small sample size (< 10 patients); (2) other publication types including conference abstracts, review articles, editorials, and letters; (3) papers irrelevant to the research question; (4) insufficient information regarding survival analysis provided for the study; and (5) overlapping study populations. When the study populations may have overlapped, we selected the publication with the largest population.

### Data extraction and quality assessment

The outcomes, study, and patient characteristics of the included studies were extracted using a standardised form. The methodological quality was appraised using the Quality in Prognostic Studies (QUIPS) tool based on key questions of prompting items and considerations for six bias domains which consist of study participation, study attrition, prognostic factor measurement, outcome measurement, study confounding, statistical analysis, and reporting [35]. Study selection, data extraction, and quality assessment were performed by two independent reviewers (S.H. and J.Y.C) with any discrepancy resolved through discussion.

### Statistical analyses

Results from the survival analyses within individual articles, including survival rates, univariate and multivariate hazard ratios (HRs), and *p* values from log-rank tests were extracted. When the Kaplan-Meier curves were provided without corresponding HRs, survival probability at each time point was extracted by means of Engauge Digitizer software (version 10.4, http://markummitchell.github.io/engauge-digitizer/) and individual patient data were reconstructed using the methodology proposed by Guyot et al. [36]. Then, Cox regression analyses were performed to derive HRs and their 95% confidence intervals (CIs); if no events were observed in one arm, Cox regression with Firth’s penalised likelihood was used.

The HRs and their 95% CIs from the univariate Cox regression analyses comparing metabolic responders and poor responders on PET scans were pooled meta-analytically using the DerSimonian-Liard method for calculating weights. If multiple HRs for a single PET parameter were provided in an individual study due to different cut-offs or regions of interest, we selected the HR with the best prognostic value for the meta-analyses. Of note, the terms ‘interim PET’ and ‘post-treatment PET’ were defined as PET studies conducted during (i.e., after one, two, or three cycles) and after NAC, respectively. Higgins *I*^2^ statistics were used to assess heterogeneity [37]. Funnel plots with Egger’s test were drawn to identify the presence of publication bias [38]. The ‘survHE’, ‘coxphf’, ‘meta’, and ‘metafor’ packages in R (R Foundation for Statistical Computing, version 3.6.0) were used for the statistical analyses.

## Results

### Study characteristics

**Fig. 1:**
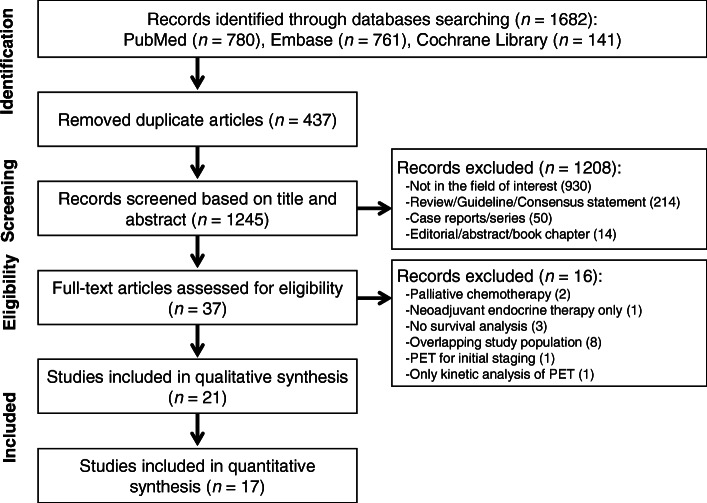
PRISMA flow chart showing the study selection process

The PRISMA study selection process is described in Fig. 1. The initial literature search yielded 1682 articles. After removing 437 duplicates, screening of the remaining 1245 titles and abstracts yielded 37 potentially eligible papers. We excluded 16 of the 37 articles for the following reasons: palliative chemotherapy (*n* = 2), neoadjuvant endocrine therapy only (*n* = 1), no survival analysis (*n* = 3), overlapping patient populations (*n* = 8), PET for baseline assessment (*n* = 1), and only kinetic analyses of dynamic PET scans (*n* = 1). Thus, 21 studies with 1630 patients were included in the qualitative synthesis [12–32]. Eleven studies were prospectively conducted, where ten were retrospective studies. For the quantitative synthesis, we included only studies where HRs for metabolic responses assessed by PET scans either during or after NAC were available. A total of 17 studies (1279 patients) were included in the quantitative synthesis [12, 16–21, 23–32]. Of note, there was one study in which Kaplan-Meier curves were separately plotted according to the therapeutic regimen [19]; these patients were incorporated into the meta-analysis as separate cohorts. Tables 1 and 2 summarise the patient and study characteristics.

**Table 1:** Study characteristics of the included studies

Author	Year	Design	Patients (*n*)	Inclusion	Neoadjuvant therapy regimen	Scanner	FDG dose (MBq)	Uptake time (min)	Median follow-up (months)
Akimoto [12]	2018	P	130	Not specified	Anthracycline + taxane-based	PET/CT	3.7/kg	60	NR
Champion [13]	2015	P	23	Inflammatory	Anthracycline ± taxane-based	PET/CT	4–5/kg	73 ± 21	76 ± 27
Chen [14]	2017	R	86	Stage II–III	Anthracycline ± taxane-based	PET/CT	259–555	60	71 (8–118)
Dunnwald [15]	2011	P	75	LABC	Anthracycline ± taxane-based	PET	218–399	60	DFS: 50 (1–156) OS: 60 (7–161)
Emmering [16]	2008	P	40	LABC	Anthracycline-based	PET	370	60–90	60
Garcia Vicente [17]	2016	P	132	Not specified	Anthracycline ± taxane-based	PET/CT	370	60	35.5^*^ (12–62)
Groheux [18]	2015	P	82	ER+/HER2−, stage II–III	Anthracycline + taxane-based	PET/CT	5/kg	60	35 (10–71)
Groheux [19]	2016	R	78	TN, stage II–III	Anthracycline + taxane-based; dose-intense anthracycline-based	PET/CT	5/kg	60	34 (3–85)
Humbert [20]	2014	P	42	HR+/HER2−, stage II–IIIA	Anthracycline ± taxane-based	PET or PET/CT	PET: 2/kg; PET/CT: 5/kg	60	64.2 (11.5–93.2)
Humbert [21]	2016	P	46	TN, Stage II–III	Anthracycline ± taxane-based	PET/CT	5/kg	60	30 (6–73)
Hyun [22]	2015	R	167	Stage II–III	Anthracycline ± taxane-based	PET/CT	5.5/kg	60	19 (3–85)
Ishiba [23]	2015	R	83	Not specified	Anthracycline ± taxane-based	PET/CT	3.7/kg	NR	50
Jung [24]	2010	P	66	Stage II–III	Anthracycline ± taxane-based	PET	370–555	60	61.5 (13.5–71.8)
Kim [25]	2016	R	139	Stage II–III	Anthracycline + taxane-based	PET/CT	5/kg	60	26.2 ± 16.1^*^
Kitajima [26]	2018	R	56	Not specified	Anthracycline ± taxane-based	PET/CT	3–4/kg	60	No recurrence: 29.1 (12.3–96.4) Recurrence: 19.2 (11.4–37.4)
Kiyoto [27]	2016	R	32	TN, stage II–III	Anthracycline ± taxane-based	PET/CT	3/kg	90	39.0 (5.8–91.2)
Kolesnikov-Gauthier [28]	2012	P	60	Non-inflammatory, M0	Anthracycline + taxane-based	PET	370	60	43 (14–68)
Lee [29]	2016	R	87	Stage II–III	Anthracycline-based	PET or PET/CT	5.5/kg	60	61 (10–107)
Lian [30]	2020	R	92	Not specified	Anthracycline ± taxane ± platinum-based	PET/CT	7.4/kg	60	No recurrence: 48.7 (20.6–84.1) Recurrence: 38.6 (11.4–82.7)
Lim [31]	2014	P	54	Stage II–III	Anthracycline + taxane-based	PET/CT	7.4/kg	60	38 (25–45)
Zucchini [32]	2013	R	60	Early, LABC, or oligometastatic	Anthracycline + taxane-based	PET/CT	5.3/kg	60–70	36.6 (8–79)

^*^mean

*CT* computed tomography, *DFS* disease-free survival, *ER* oestrogen receptor, *HR* hormone receptor. *HER2* human epidermal growth factor receptor 2, *LABC* locally advanced breast cancer, *OS* overall survival, *P* prospective, *PET* positron emission tomography, *R* retrospective, *TN* triple-negative

**Table 2:** Patient characteristics in the included studies

Author	Mean age (range)	Initial stage (%)	Histology (ductal/lobular, %)	Grade (I/II/III, %)	Receptor phenotypes (ER+/PR+/HER2+, %)	Subtypes (luminal A/B/HER2/TN, %)	pCR (%)
Akimoto [12]	53.9	I/II/III: 8/73/19	NR	4/28/67 Unknown: 2	69/57/28	ER+/HER2−: 53 HER2+: 28 TN: 19	23
Champion [13]	51 ± 12.7	all T4d, M0	95/0	9/30/61	HR+:43 HER2+: 22	HR+/HER2−: 48 HR−/HER2+: 22 TN: 30	22
Chen [14]	51^†^	II/III: 14/86	NR	NR	56/37/28	ER+/HER2−: 43 HER2+: 28 TN: 29	17
Dunnwald [15]	NR	T1/2/3/4: 3/21/57/19 N0/1/2/3: 19/61/17/3	92/8	NR	55/45/26	NR	28
Emmering [16]	48^†^ (29–63)	IIB: 15 IIIA/B/C: 43/30/13	70/13	NR	NR	NR	20
Garcia Vicente [17]	52.6 ± 12.7 (25–80)	NR	92/8	NR	NR	10/55/12/23	NR
Groheux [18]^*^	50.4 ± 11.7 (30–82)	T1/2/3/4: 2/39/39/20 N0/1/2/3: 39/48/10/3	90/7	6/68/26	100/67/0	ER+/HER2−: 100	5
Groheux [19]	51^†^ (27–78)	IIA/B: 27/23 IIA/B/C: 23/22/5	94/0	0/10/88	0/0/0	TN: 100	37
Humbert [20]	NR	T1–2/3: 88/12 N0/1–2: 38/62	90/10	5/80/15 Unknown: 2	98/91/0	HR+/HER2−: 100 Luminal A/B: 5/76	2
Humbert [21]	46^†^ (26–85)	IIA/B: 35/30 IIIA/B/C: 9/4/22	98/2	0/18/78	0/0/0	TN: 100	43
Hyun [22]	44 (22–68)	II/III: 32/68	95/2	NR	HR+: 51 HER2+: 27	HR+/HER2−: 40 HR−/HER2+: 16 TN: 33	17
Ishiba [23]	54^†^ (30–75)	II/III: 82/18	95/1	45/24/24 Unknown: 7	NR	luminal A and B 65 luminal HER2 7 HER2: 12 TN 16	17
Jung [24]	44^†^ (21–64)	II/III: 56/44	NR	NR	56/36/41	NR	T/N^‡^: 15/29
Kim [25]	46.5 (27–72)	II/III: 80/20	100/0	21/44/35	NR	HR+/HER2–55 HER2+: 27 TN: 18	16
Kitajima [26]	53.6 ± 12.4 (29–77)	I/II/III: 7/48/45	96/0	12/11/32 Unknown: 45	50/38/45	14/39/20/27	34
Kiyoto [27]	54^†^ (31–71)	IIA/B: 16/34 IIIA/B/C: 22/3/25	94/0	16/37/47	0/0/0	TN:100	22
Kolesnikov-Gauthier [28]	49 ± 9 (30–70)	T2/3: 60/37 N0/1/2: 58/37/3	97/3	5/42/27 Unknown: 27	53/37/20	NR	22
Lee [29]	46.1 (26–73)	T1/2/3/4: 14/70/10/6 N1/2/3: 61/14/25	NR	11/33/22 Unknown: 33	41/33/48	25/20/31/24	20
Lian [30]	48.1 (28–76)	II/III 74/26	100/0	NR	58/50/44	1/58/20/21	36
Lim [31]	48^†^ (26–68)	T1/2/3/4: 9/41/33/17 N0/1/2/3: 4/20/54/13	93/4	NR	54/74/35	NR	NR
Zucchini [32]	49^†^ (31–72)	II/III/IV: 50/38/12	NR	NR	NR	ER+/HER2−: 52 HER2+: 23 TN: 25	22

^*^Age and clinical stages were only available for whole study population, whereas survival analysis was performed in a subset of subjects

^†^Median

^‡^T and N represent pCR rate in the primary tumour and axillary node, respectively

*ER* oestrogen receptor, *HR* hormone receptor, *HER2* human epidermal growth factor receptor 2, *NR* not reported, *pCR* pathological complete response, *PR* progesterone-receptor, *TN* triple-negative

### Quality assessment

**Fig. 2:**
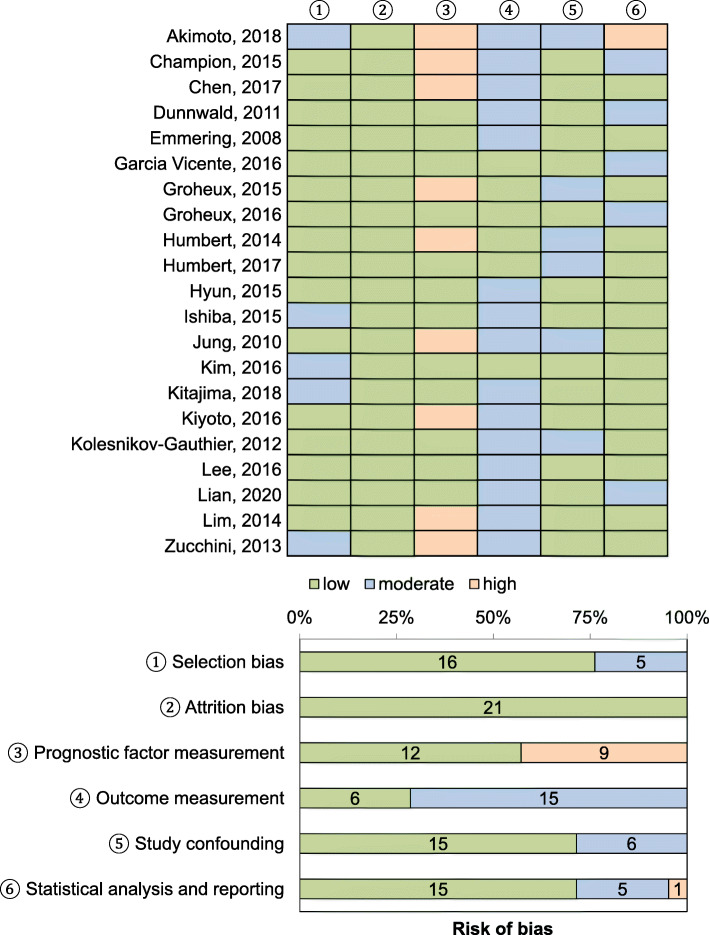
Quality assessment using the QUIPS tool

The quality assessment performed using the QUIPS tool is illustrated in Fig. 2. The specific number of studies at risk of bias and reasons were as follows: Five studies presented a moderate risk in selection of participants because of the retrospective study designs, lack of clarity regarding whether the patients were enrolled in a consecutive manner, and/or the unclear inclusion and exclusion criteria [12, 23, 25, 26, 32]. All studies were rated as having a low risk of attrition bias. For prognostic factor measurement, nine studies had a high risk of bias due to the use of data-dependent cut-off values [12–14, 18, 20, 24, 27, 31, 32]. Regarding outcome measurements, fifteen studies had a moderate risk of bias because the definition or methods for measuring disease recurrence were unclear [12–16, 22–24, 26–32]. Six studies presented a moderate risk of confounding bias as no adjustment for potential confounders was performed [12, 18, 20, 21, 24, 28]. With regard to statistical analysis domains, five studies had a moderate risk of bias as it was unclear which variables were incorporated into the multivariate analyses, or too many variables were included in the multivariate analyses considering the number of patients in the study population [13, 15, 17, 19, 30]. One study had a high risk of bias in the statistical analyses due to possible selective reporting, as the results of survival analysis were provided for only a subset of the study population, and not the whole population [12].

### Qualitative synthesis

The outcomes of the included articles are summarised in Table 3. PET scanning for evaluation of the patients’ response to NAC was performed during (interim PET) or after treatment (post-treatment PET) in 12 and 10 studies, respectively. The most widely evaluated PET parameter was the percent reduction of maximum standardised uptake value (SUVmax) compared to baseline SUVmax (%ΔSUVmax), followed by SUVmax and complete metabolic response (CMR) on interim or post-treatment PET scans. Of note, %ΔSUVmax is defined as (SUVmax at baseline PET − SUVmax at interim or post-treatment PET) / SUVmax at baseline PET × 100%. The CMR in the included studies was defined as negative FDG uptake [21], or according to the European Organisation for Research and Treatment of Cancer (EORTC) or Positron Emission Tomography Response Criteria in Solid Tumours (PERCIST) criteria [17, 26, 30]. Regarding the timing of interim assessment, PET or PET/CT were performed after two cycles of NAC in five studies [17–19, 30, 32], one cycle in four [20, 21, 28, 31], and three cycles in two studies [13, 29]. Specifically, higher %ΔSUVmax at interim evaluation was significantly associated with longer DFS in seven of ten studies [13, 17–21, 28, 29, 31, 32], and longer OS in two of six studies [13, 17, 20, 21, 28, 29]. In addition, CMR and lower SUVmax on interim PET scan was significantly associated with longer DFS in one of two studies [17, 30], and one study [18], respectively. Regarding post-treatment PET scans, %ΔSUVmax was a significant prognostic factor for DFS in four of six studies [12, 13, 17, 22, 25, 27]. Lower SUVmax was significantly associated with longer DFS in all five studies [12, 17, 22, 23, 25]. CMR was correlated with better DFS in three studies [16, 17, 26]; however, CMR was no longer statistically significant after completion of multivariate analyses of the data from two of them [17, 26]. Two studies reported that metabolic tumour volume and total lesion glycolysis and their reduction rates on post-treatment PET scans were significant predictors for better DFS [22, 25].

**Table 3:** Summary of outcomes in the included studies

Author	PET timing	Parameter	DFS	OS
Akimoto [12]	After completion	%ΔSUVmax > 80	N/S for TN or HER2+	NR
		SUVmax ≤ 1.3	*P* = 0.026 for TN or HER2+	NR
Champion [13]	After 3 cycles	%ΔSUVmax	N/S	N/S
	After completion	%ΔSUVmax > 72	*P* = 0.05* Adjusted *P* = 0.01*	N/S
Chen [14]	During or after	Mid- or post-SUVmax^†^	HR = 1.13 (1.06–1.21) Adjusted HR = 1.09 (1.01–1.17)	HR = 1.16 (1.08–1.24) Adjusted HR = 1.14 (1.06–1.23)
		Mid- or post-SUVmax < 2.5	HR = 0.28 (0.13–0.62)	HR = 0.25 (0.11–0.60)
		%ΔSUVmax^†^	N/S	N/S
Dunnwald [15]	At mid-therapy	Log2(SUVpeak)^†^	N/S	HR = 1.96 (1.14–3.34) Adjusted HR = N/S
		Δ%SUVpeak^†^	N/S	HR = 0.72 (0.54–0.96) Adjusted HR = N/S
Emmering [16]	After completion	CMR	HR = 0.24 (0.08–0.79) Adjusted HR = 0.28 (0.08–0.96)	N/S
Garcia Vicente [17]	After 2 cycles	CMR-tumour	N/S	N/S
		%Δtumour-SUVmax ≥ 62	N/S	N/S
		CMR-lymph node	N/S	N/S
	After completion	CMR-tumour	N/S	N/S
		CMR-lymph node	*P* = 0.003 Adjusted *P* = N/S	*P* = 0.016 Adjusted *P* = N/S
		Tumour-SUVmax	< 1.05: HR = 0.06 (0.01–0.47)	< 1.15: N/S
		Lymph node-SUVmax	< 1.30: N/S	< 0.40: N/S
		%Δtumour-SUVmax	≥ 74: N/S	≥ 84: N/S
Groheux [18]	After 2 cycles	SUVmax < 7.4	*P* = 0.011	NR
		%ΔSUVmax ≥ 12	*P* = 0.033	NR
		TLG < 30.5	*P* = 0.017	NR
		%ΔTLG ≥ 51	*P* < 0.001	NR
Groheux [19]	After 2 cycles	%ΔSUVmax^†^	HR = 0.86 (0.78–0.94) Adjusted *P* = 0.004	NR
		%ΔSUVmax	*P* = 0.021 and *P* = 0.028^‡^	NR
Humbert [20]	After 1 cycle	%ΔSUVmax ≥ 16	HR = 0.19 (0.06–0.64)	HR = 0.09 (0.02–0.54)
Humbert [21]	After 1 cycle	%ΔSUVmax ≥ 50%	N/S	N/S
Hyun [22]	After completion	Log2(SUVmax)^†^	HR = 1.86 (1.38–2.51) Adjusted HR = 1.51 (1.04–2.19)	NR
		%ΔSUVmax^†^	HR = 0.98 (0.97–0.99) Adjusted HR = 0.99 (0.98–1.00)	NR
		Log2(MTV)^†^	HR = 1.26 (1.15–1.38) Adjusted HR = 1.14 (1.01–1.27)	NR
		%ΔMTV^†^	HR = 0.99 (0.99–1.00) Adjusted HR = 1.00 (0.99–1.00)	NR
Ishiba [23]	After completion	SUVmax ≤ 1.7	*P* = 0.004 Adjusted *P* = 0.014	*P* = 0.01 Adjusted *P* = 0.029
Jung [24]	After completion	Δ%SUVpeak ≥ 84.8	*P* = 0.04 Adjusted *P* = N/S	NR
Kim [25]	After completion	SUVmax^†^	HR = 1.20 (1.12–1.28)	NR
		MTV^†^	HR = 1.02 (1.01–1.03)	NR
		TLG^†^	HR = 1.00 (0.99–1.00)	NR
		%ΔSUVmax^†^	HR = 0.99 (0.98–0.99)	NR
		%ΔMTV^†^	HR = 1.00 (0.99–1.00) Adjusted HR = 0.99 (0.98–1.00)	NR
		%ΔMTV > 90.7	HR = 0.39 (0.19–0.79)	NR
		%ΔTLG^†^	HR = 0.99 (0.99–1.00)	NR
Kitajima [26]	After completion	CMR	HR = 0.15 (0.02–0.70) Adjusted HR = N/S	NR
Kiyoto [27]	After completion	%ΔSUVmax > 15.9	HR = 0.18 (0.05–0.88)	NR
Kolesnikov-Gauthier [28]	After 1 cycle	%ΔSUVmax > 15	4-year DFS: 85% vs. 44%, *P* = 0.008	N/S
Lee [29]	After 3 cycles	%ΔSUVmax > 66.4	*P* < 0.001	*P* = 0.009
		%ΔSUVmax^†^	HR = 0.97 (0.95–0.98) Adjusted HR = 0.97 (0.95–0.99)	HR = 0.98 (0.96–0.99) Adjusted HR = 0.97 (0.95–0.99)
Lian [30]	After 2 cycles	CMR	HR = 0.17 (0.04–0.73) Adjusted HR = 0.04 (0.00–0.42)	NR
Lim [31]	After 1 cycle	%ΔSUVmax > 41	*P* < 0.001 Adjusted HR = 0.13 (0.03–0.49)	NR
Zucchini [32]	After 2 cycles	%ΔSUVmax > 50	N/S for all *P* = 0.049 for ER+/HER2−	NR

*Distant metastasis-free survival

^†^As continuous variables

^‡^For two cohorts with different NAC regimens

%ΔSUVmax was defined as (SUVmax at baseline PET − SUVmax at interim or post-treatment PET)/SUVmax at baseline PET × 100%

*CMR* complete response, *DFS* disease-free survival, *ER* oestrogen receptor, *HER2* human epidermal growth factor receptor 2, *HR* hazard ratio, *MTV* metabolic tumour volume, *NR* not reported, *N/S* not significant, *OS* overall survival, *SUV* standardised uptake value, *TLG* total lesion glycolysis, *TN* triple-negative

The five studies exclusively included specific hormonal subtype of either TN or HR+/HER2−. Of two studies for HR+/HER2− subtype [18, 20], higher %ΔSUVmax on interim PET assessment was significantly associated with better DFS and OS. Likewise in three studies for TN subtype [19, 21, 27], higher %ΔSUVmax on interim PET scans or post-treatment PET scans was a significant predictor for longer DFS.

### Quantitative synthesis

**Fig. 3:**
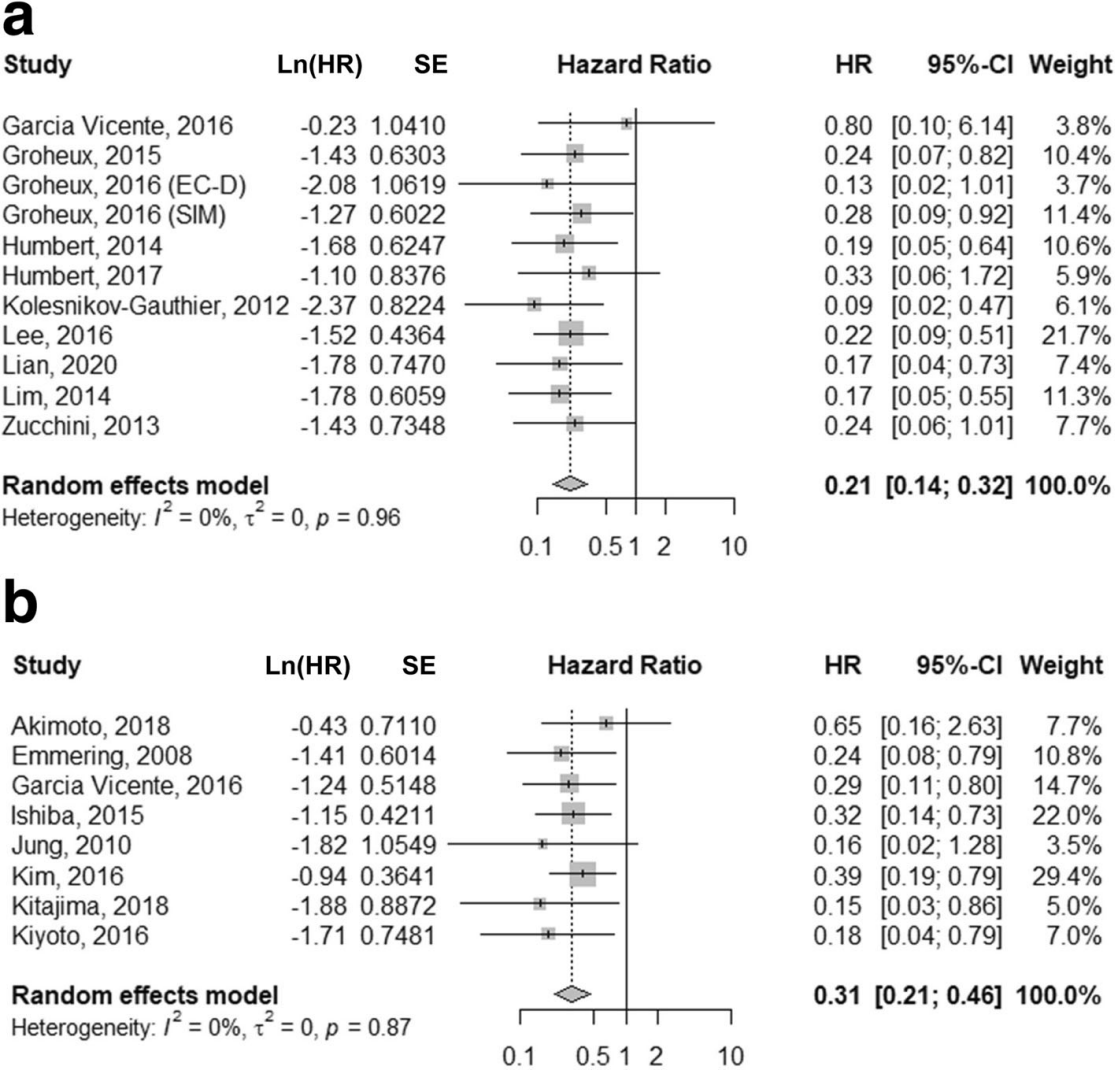
Forest plots showing the pooled HRs of the PET response on interim (**a**) and post-treatment (**b**) ^18^F-FDG PET scans for disease-free survival

**Fig. 4:**
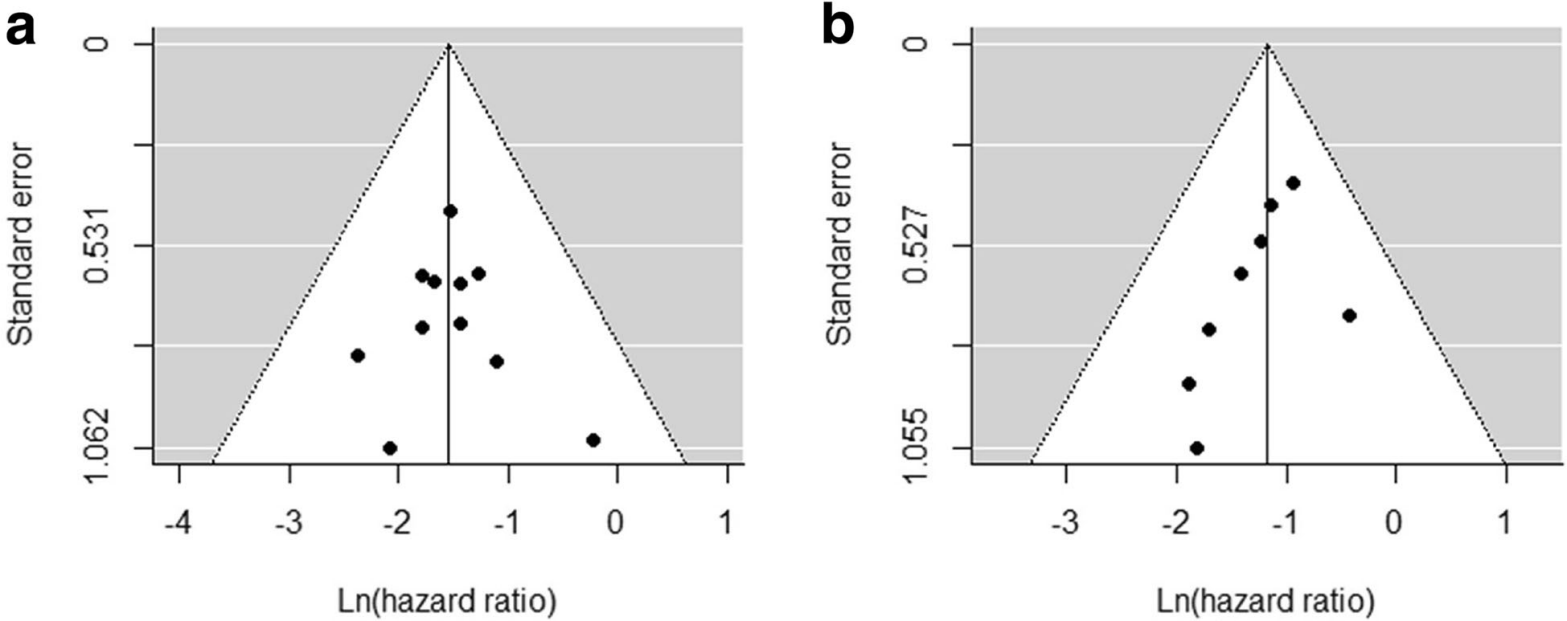
Funnel plots of studies assessing the PET response on interim (**a**) and post-treatment (**b**) ^18^F-FDG PET scans for disease-free survival

Meta-analytical pooling of HRs for interim and post-treatment PET analyses on DFS and OS was performed. With regard to the influence of metabolic responses on DFS, the pooled HR for interim PET scans was 0.21 (95% CI, 0.14–0.32; Fig. 3a) with no heterogeneity (*I*^2^ = 0%). No publication bias was shown in the funnel plot and Egger’s test (*P* = 0.8654; Fig. 4a). PET response analyses performed after one, two, and three cycles of NAC showed comparable prognostic values for DFS with pooled HRs of 0.18 (95% CI, 0.09–0.35), 0.25 (95% CI, 0.14–0.45), and 0.22 (95% CI, 0.09–0.51), respectively (*P* for subgroup difference = 0.7661). The pooled HR for metabolic responses on post-treatment PET/CT was 0.31 (95% CI, 0.21–0.46; Fig. 3b). No heterogeneity was found (*I*^2^ = 0%), and no publication bias was present (*P* = 0.3199; Fig. 4b). No statistical difference was found between the pooled HRs of interim and post-treatment PET response analyses (*P* = 0.1942). For studies using combined PET/CT, the pooled HRs for interim and post-treatment PET/CT were 0.23 (95% CI, 0.15–0.37) and 0.30 (95% CI, 0.20–0.43), respectively. The results of subgroup analyses according to PET response parameters are provided in Table 4.

**Table 4:** Subgroup analysis according to PET timing and parameters

Outcomes	PET timing	PET parameter	Studies (*n*)	Pooled hazard ratios	95% confidence interval	*I*^2^ (%)	*P* for subgroup difference
Disease-free survival	Interim	%ΔSUVmax	9	0.20	0.13–0.31	0	0.8539
		SUVmax	1	0.24	0.07–0.82	NA	
		CMR	2	0.31	0.07–1.37	32	
	Post-treatment	%ΔSUVmax	2	0.16	0.05–0.48	0	0.5821
		%ΔSUVpeak	1	0.16	0.02–1.28	NA	
		%ΔMTV	1	0.39	0.19–0.79	NA	
		ΔSUVmax	2	0.38	0.19–0.78	0	
		CMR	3	0.25	0.12–0.50	0	
Overall survival	Interim	%ΔSUVmax	3	0.20	0.09–0.44	0	0.7303
		CMR	1	0.34	0.02–6.64	NA	
	Post-treatment	SUVmax	1	0.30	0.11–0.81	NA	0.7373
		CMR	2	0.24	0.10–0.59	0	

*CMR* complete response, *MTV* metabolic tumour volume, *NA* not applicable, *SUV* standardised uptake value

Among nine studies assessing the prognostic value of %ΔSUVmax for DFS on interim PET scans, six studies included patients with initial clinical stage II–III cancers; %ΔSUVmax was a significant predictor of DFS in Stage II–III breast cancer with a pooled HR of 0.21 (95% CI, 0.13–0.34; Additional file 2: Fig. S1). Of these, there were four studies which included either ER+/HER2− or triple-negative breast cancer population; %ΔSUVmax was also a significant prognostic factor in stage II–III ER+/HER2− or triple-negative breast cancer (pooled HRs = 0.20 [95% CI, 0.08–0.47] and 0.26 [95% CI, 0.11–0.61], respectively). Meta-regression analyses were performed according to clinical variables (including age, stage, histologic type and grade, receptors, subtypes, and pathological complete response) for these nine studies, and no variable was found to significantly influence the HRs (Additional file 3: Table S1).

**Fig. 5:**
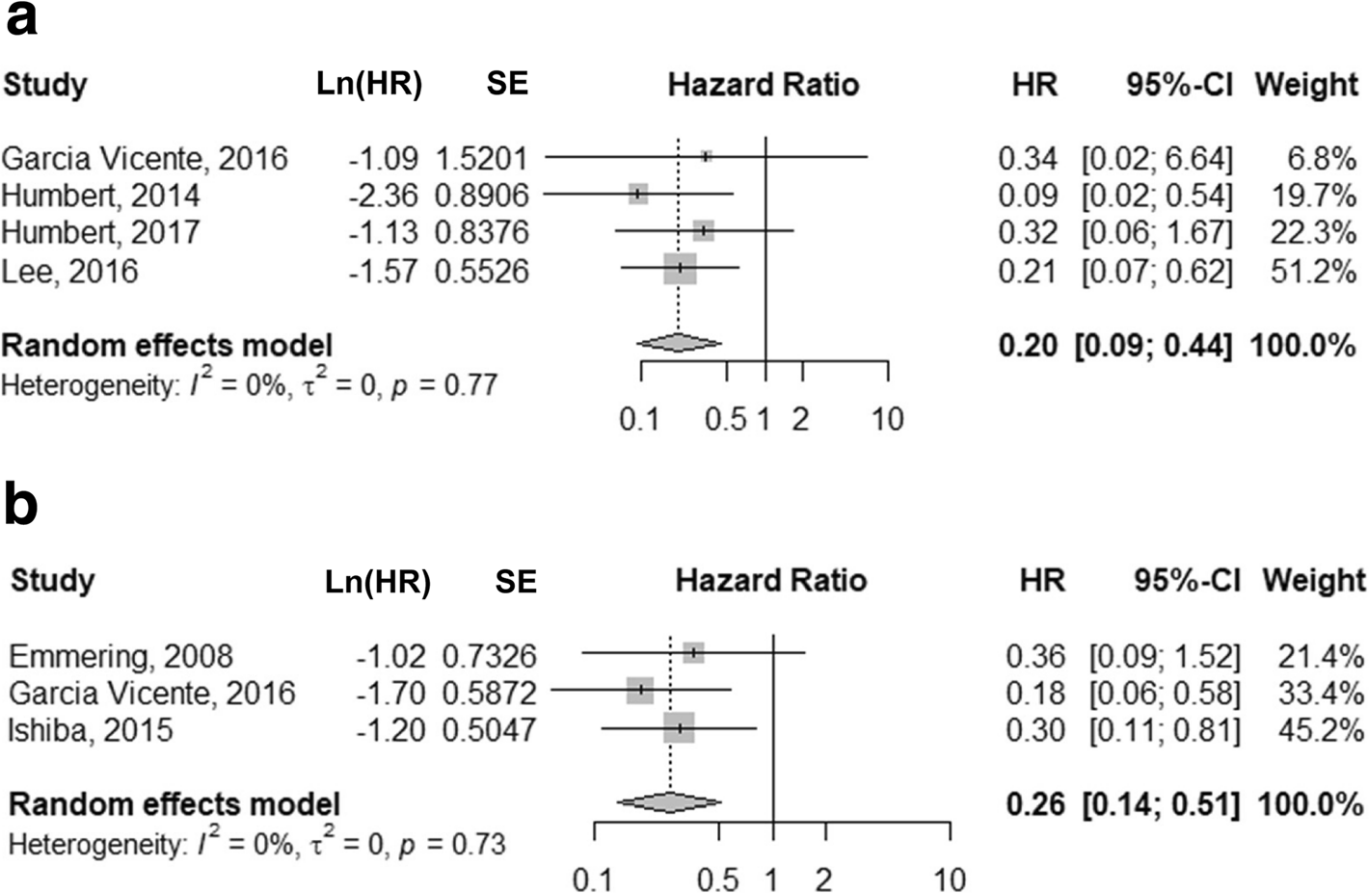
Forest plots showing the pooled HRs of the PET response on interim (**a**) and post-treatment (**b**) ^18^F-FDG PET scans for overall survival

With regard to the influence of metabolic response on OS, the pooled HR for the interim PET scans was 0.20 (95% CI, 0.09–0.44; Fig. 5a), and no heterogeneity was present (*I*^2^ = 0%). The pooled HRs for the metabolic response on post-treatment PET scans was 0.26 (95% CI, 0.14–0.51; Fig. 5b). No heterogeneity was found (*I*^2^ = 0%). No statistical difference was found between the pooled HRs of interim and post-treatment PET response analyses (*P* = 0.6137). Publication bias could not be evaluated because of the limited number of included studies. No statistical difference was found upon subgroup analyses in accordance with PET parameters (Table 4), although the paucity of studies limited the statistical power. For studies using combined PET/CT, the pooled HRs for interim and post-treatment PET/CT were 0.33 (95% CI, 0.08–1.37) and 0.24 (95% CI, 0.11–0.51), respectively.

## Discussion

In this meta-analysis, we assessed the prognostic value of ^18^F-FDG PET or PET/CT for evaluation of the response to treatment in breast cancer patients. Our major findings were as follows; (1) PET metabolic response during and after NAC is a significant predictor of disease recurrence and death; (2) more evidence is available regarding PET scans for prediction of DFS than OS (18 and 7 studies included in meta-analyses for DFS and OS, respectively); (3) %ΔSUVmax was the most frequently evaluated response parameter in both interim and post-treatment PET scans; (4) no differences were found between the prognostic values of interim and post-treatment PET scans in term of both DFS and OS; (5) regarding the timing of the interim assessment, PET is mostly commonly performed after 2 cycles (followed by PET after 1 cycle) and yielded significant prognostic values; and (6) in more than half of the included studies, the cut-off values for each PET parameter were data-dependent and therefore differed greatly across studies.

Notably, a metabolic response identified on PET scans is significantly associated with the pathological response in surgical specimens after NAC [7–9], a well-established predictor of patient outcomes [39]. In addition, in clinical practice, it is plausible that ^18^F-FDG PET or PET/CT is highly likely to have an incremental prognostic value on pathological response of breast cancer given (1) PET scans enable early assessment of patient responses to NAC, which may support decisions to cease ineffective treatment and select alternative treatment options, whereas pathological response can only be assessed after completion of surgical resection; (2) twelve of 15 included studies in which either multivariate Cox regression analyses or subgroup analyses were performed reported the metabolic response as having independent prognostic significance to pathological response [13–17, 19, 22–29, 31]. We could not pool HRs from multivariate Cox regression in the meta-analysis because variables included in the models differed widely across the studies that would directly affect the values of HR; (3) the results of our meta-regression indicated the pathological complete response did not influence the pooled HR, which may indirectly support the independent prognostic role of the metabolic response.

Upon thorough inspection of the clinical characteristics of the included studies, our study population mainly consisted of Stage II–III breast cancer patients, which was consistent with the types of patients who typically receive NAC [33]. We also found higher proportions of patients with HER2+ and triple-negative subtypes in the included studies (HER2+ regardless of hormonal receptor status: 26 [402/1558]; and TN: 28 [374/1357]) compared to general breast cancer population referred to cancer statistics published by the US National Cancer Institute: the prevalence of the HER2+ and triple-negative subtypes was 14% and 10%, respectively [40]. HER2+ or triple-negative subtypes are aggressive subtypes, with patients typically presenting with higher FDG uptake at baseline. Therefore, these subtypes of breast cancer are promising targets for evaluation of the metabolic response using ^18^F-FDG PET or PET/CT [33]. In addition, our analyses indicated that a metabolic response on interim PET also has prognostic significance in the ER+/HER2− group [18, 20], a subtype in which MRI is of limited utility for evaluation of patient responses to NAC [4, 5].

The studies included in our qualitative synthesis varied widely in terms of PET timing, and PET criteria for defining a metabolic response. %ΔSUVmax, the percent reduction of SUVmax between the baseline and interim or post-treatment PET scans, is the most frequently evaluated parameter and is associated with disease recurrence and survival. This ‘ratio’ has the advantage that it may offset the potential effect of noise, reconstruction, image sampling, and smoothing on SUVmax, as long as the PET scans at baseline and during or after NAC are performed using the same machine and protocols; otherwise, it may limit the applicability of results across PET facilities [41].

There were a comparable number of studies and prognostic significance regarding interim vs. post-treatment PET. As it can allow early response evaluation and subsequent modification in treatment, interim PET scans may have better clinical values than post-treatment PET. Regarding specific timing of interim PET, there were no apparent differences between the prognostic values of interim PET assessments performed at different times during NAC; however, the number of studies was insufficient to assess statistical significance. We found that in the majority of studies addressing interim PET scans the response was assessed after 1–2 cycles of NAC, and evidence of their prognostic value was found. Moreover, the better predictive values for pathological response when performing PET scans after 1–2 cycles of NAC (compared to after 3 cycles of NAC) were reported in previous meta-analyses [7, 10]. Given early assessment of response to NAC is important for timely modification of the therapeutic strategy, it might be advisable for interim PET to be performed after 1–2 cycles.

There were several limitations of our study. First, a substantial portion of the included studies were performed retrospectively. Second, there was considerable heterogeneity of hormonal subtype of tumour, PET scan timing, and response parameters among the studies. Therefore, caution is required when considering the applicability of our pooled estimates. Third, approximately half of the included studies used data-dependent cut-off values for the assessment of PET parameters (i.e., optimal cut-off on receiver operating characteristics analysis for predicting pathological response) which may overestimate the prognostic values of ^18^F-FDG PET or PET/CT. Fourth, the number of studies included for meta-analysis of OS was small, though the pooled HR was statistically significant. However, DFS was regarded as a valid surrogate for OS which requires long-term follow-up for the assessment of efficacy [42].

## Conclusions

A metabolic response to NAC as detected by ^18^F-FDG PET or PET/CT is a significant prognostic factor in terms of DFS and OS. Meta-analytically pooled HRs for DFS nor OS were not significantly different for interim or post-treatment PET scans. %ΔSUVmax, defined as the percent reduction of SUVmax compared with that obtained from the baseline PET, is the most widely evaluated PET response parameter. For the interim assessment of patient responses to NAC, PET scans were commonly performed after 1–2 cycles of NAC and provided significant prognostic values. Evaluation of the metabolic response to NAC may be helpful not only in HER2+ or triple-negative subtypes which are known to be FDG-avid, but also in hormone receptor-positive tumours. These results suggest that ^18^F-FDG PET or PET/CT may provide accurate risk stratification of breast cancer patients and support risk-adapted therapeutic management based on metabolic response in clinical practice or trials.

## Supplementary information

**Additional file 1.** The queries and results of electronic searches of the PubMed, Embase, and Cochrane Library databases. We provided queries and results of electronic searches of the PubMed, Embase, and Cochrane Library databases as tables.

**Additional file 2: Figure S1.** Forest plots of studies assessing the HRs of %ΔSUVmax at the interim evaluation for disease-free survival in Stage II–III breast cancer. We provided forest plots supporting the pooled HRs for the influence of %ΔSUVmax on disease-free survival in stage II–III breast cancer patients at the interim evaluation. (PPTX 78 kb)

**Additional file 3: Table S1.** Meta-regression analyses of nine studies where hazard ratios for the influence of %ΔSUVmax on disease-free survival at interim PET scan were available. We provided detailed results of meta-regression analyses including regression coefficients and *P* values.

## Data Availability

Not applicable (The current study was performed based on published literature and no datasets were generated.)
