# Supplementary material for: Prognostic value of 18F-FDG PET and PET/CT for assessment of treatment response to neoadjuvant chemotherapy in breast cancer: a systematic review and meta-analysis
Source: Breast Cancer Res. 2020 Oct 31;22:119. doi: 10.1186/s13058-020-01350-2 (PMC7603771; doi:10.1186/s13058-020-01350-2)
Supplement: Supplementary file 1 — Additional file 1. The queries and results of electronic searches of the PubMed, Embase, and Cochrane Library databases. We provided queries and results of electronic searches of the PubMed, Embase, and Cochrane Library databases as tables. [file 13058_2020_1350_MOESM1_ESM.docx]

**Additional file 1.** The queries and results of electronic search on PubMed, Embase, and Cochrane Library.

PubMed

| Date: 2020.06.04 | | | |
| --- | --- | --- | --- |
| PICOS | # | Search queries | Articles # |
| P-KQ1 | **1** | ((Breast Neoplasms[MeSH Terms]) OR (breast AND (tumor OR tumour OR cancer OR carcinoma OR malignan* OR neoplasm*))) ALL | 420,576 |
| I | **2** | ((Positron-Emission Tomography[MeSH Terms]) OR (“positron-emission tomography” OR “positron emission tomography” OR PET OR PET?CT OR PET/CT OR PET-CT OR PET?MRI OR PET?MR OR PET/MRI OR PET/MR OR PET-MRI OR PET-MR)) ALL | 132,796 |
|  | **3** | ((Neoadjuvant Therapy[MeSH Terms]) OR (neoadjuvant OR preoperative OR pre-operative OR early OR interim)) ALL | 1,914,238 |
| I-KQ1 | **4** | #2 AND #3 | 19.179 |
| P&I KQ1 | **5** | #1 AND #4 | 1,251 |
| O | **6** | ((Prognosis[MeSH Terms]) OR (Survival Analysis[MeSH Terms]) OR (prognos* OR predict* OR survival OR outcome)) ALL | 5,516,202 |
| KQ1 limit | **7** | #5 AND #6 | 831 |
| case report | **8** | Case Reports [Publication Type] |  |
| Final KQ1 | **9** | #7 NOT #8 | **780** |

Embase

| Date: 2020.06.04 | | | |
| --- | --- | --- | --- |
| PICOS | # | Search queries | Articles # |
| P-KQ1 | **1** | "breast tumor"/mj OR (breast AND (tumor OR tumour OR cancer OR carcinoma OR malignan* OR neoplasm*)):ti,ab,kw | 523,367 |
| I | **2** | "positron emission tomography"/exp OR (“positron-emission tomography” OR “positron emission tomography” OR PET OR “PET CT” OR PET*CT OR PET-CT OR “PET MRI” OR “PET MR” OR PET*MRI OR PET*MR OR PET-MRI OR PET-MR):ti,ab,kw | 245,323 |
|  | **3** | “neoadjuvant therapy”/exp OR (neoadjuvant OR preoperative OR pre-operative OR early OR interim):ti,ab,kw | 2,477,400 |
| I-KQ1 | **4** | #2 AND #3 | 39,497 |
| P&I KQ1 | **5** | #1 AND #4 | 2,701 |
| O | **6** | "prognosis"/exp OR "survival"/exp OR (prognos* OR predict* OR survival OR outcome):ti,ab,kw | 5,006,267 |
| KQ1 limit | **7** | #5 AND #6 | 1,515 |
| Final KQ1 | **8** | #7 AND ('article'/it OR 'article in press'/it OR 'review'/it) | **761** |

Cochrane Library

| Date: 2020.06.04 | | | |
| --- | --- | --- | --- |
| PICOS | # | Search queries | Articles # |
| P | **1** | (breast AND (tumor OR tumour OR cancer OR carcinoma OR malignan* OR neoplasm*)) | 37,821 |
|  | **2** | MeSH descriptor: [Breast Neoplasms] explode all trees | 12,761 |
| P-KQ1 | **3** | #1 OR #2 | 37,821 |
| I | **4** | (“positron emission tomography” OR PET OR “PET CT” OR PET*CT OR PET-CT OR “PET MRI” OR “PET MR” OR PET*MRI OR PET*MR OR PET-MRI OR PET-MR) | 7,958 |
|  | **5** | MeSH descriptor: [Positron-Emission Tomography] explode all trees | 959 |
|  | **6** | #4 OR #5 | 7,958 |
|  | **7** | (neoadjuvant OR preoperative OR pre-operative OR early OR interim) | 164,405 |
|  | **8** | MeSH descriptor: Neoadjuvant Therapy | 1,109 |
|  | **9** | #7 OR #8 | 164,405 |
| I KQ1 | **10** | #6 AND #9 | 1,941 |
| P&I KQ1 | **11** | #3 AND #10 | 180 |
| O | **12** | (prognos* OR predict* OR survival OR outcome) | 597,473 |
|  | **13** | MeSH descriptor: Prognosis | 149,852 |
|  | **14** | MeSH descriptor: Survival Analysis | 20,143 |
|  | **15** | #12 OR #13 OR #14 | 600,684 |
| KQ1 limit | **16** | #11 AND #15 | 150 |
| Final KQ1 | **17** | #16 AND Cochrane Review, Trial | **141** |
