# Supplementary figures and images for: Prognostic value of 18F-FDG PET and PET/CT for assessment of treatment response to neoadjuvant chemotherapy in breast cancer: a systematic review and meta-analysis
Source: Breast Cancer Res. 2020 Oct 31;22:119. doi: 10.1186/s13058-020-01350-2 (PMC7603771; doi:10.1186/s13058-020-01350-2)

## Slide 1
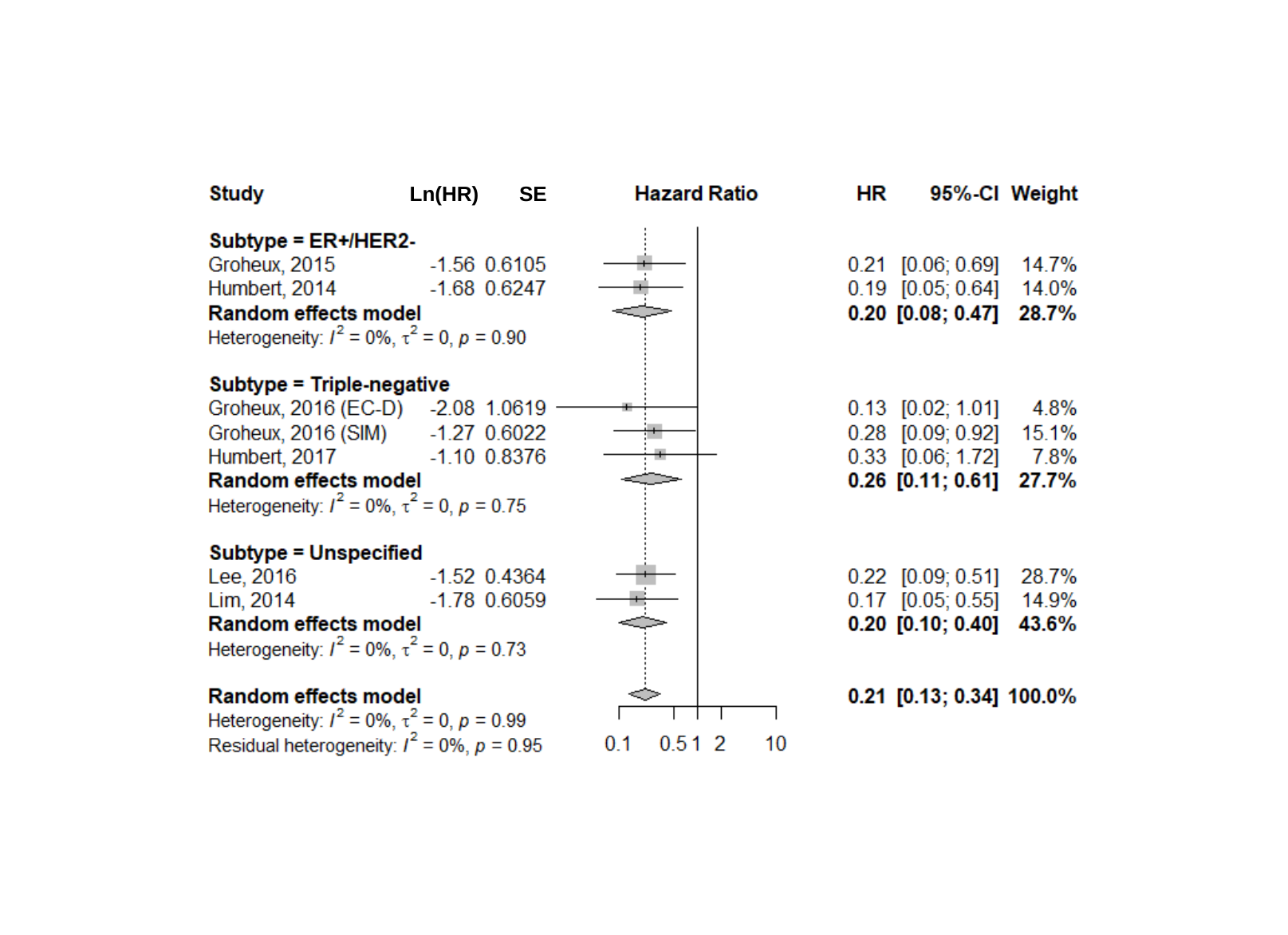

Ln(HR) SE

Supplement: Supplementary file 2 — Additional file 2: Figure S1. Forest plots of studies assessing the HRs of %ΔSUVmax at the interim evaluation for disease-free survival in Stage II–III breast cancer. We provided forest plots supporting the pooled HRs for the influence of %ΔSUVmax on disease-free survival in stage II–III breast cancer patients at the interim evaluation. (PPTX 78 kb) [file 13058_2020_1350_MOESM2_ESM.pptx]
