# Supplementary material for: Prognostic value of 18F-FDG PET and PET/CT for assessment of treatment response to neoadjuvant chemotherapy in breast cancer: a systematic review and meta-analysis
Source: Breast Cancer Res. 2020 Oct 31;22:119. doi: 10.1186/s13058-020-01350-2 (PMC7603771; doi:10.1186/s13058-020-01350-2)
Supplement: Supplementary file 3 — Additional file 3: Table S1. Meta-regression analyses of nine studies where hazard ratios for the influence of %ΔSUVmax on disease-free survival at interim PET scan were available. We provided detailed results of meta-regression analyses including regression coefficients and P values. [file 13058_2020_1350_MOESM3_ESM.docx]

**Table S1.** Meta-regression analyses of nine studies where hazard ratios for the influence of %ΔSUVmax on disease-free survival at the interim PET scan were available

| Variables | Studies (*n*) | Regression coefficient | 95% confidence interval | *P* |
| --- | --- | --- | --- | --- |
| Mean age (y) | 8 | -0.0200 | -0.2494–0.2094 | 0.8641 |
| Initial stage ≥III (%) | 4 | -0.0222 | -0.1329–0.0885 | 0.6941 |
| Ductal histology (%) | 7 | 0.0281 | -0.1253–0.1815 | 0.7196 |
| Grade III (%) | 7 | 0.0048 | -0.0120–0.0217 | 0.5747 |
| ER+ phenotype (%) | 7 | -0.0027 | -0.0149–0.0096 | 0.6681 |
| PR+ phenotype (%) | 7 | -0.0039 | -0.0178–0.0099 | 0.5795 |
| HER2+ phenotype (%) | 8 | -0.0019 | -0.0227–0.0190 | 0.8609 |
| ER+/HER2- phenotype (%) | 6 | -0.0026 | -0.0148–0.0096 | 0.6800 |
| Luminal A subtype (%) | 5 | -0.0039 | -0.0510–0.0432 | 0.8707 |
| Luminal B subtype (%) | 5 | -0.0040 | -0.0237–0.0157 | 0.6895 |
| HER2-enriched subtype (%) | 6 | -0.0010 | -0.0349–0.0329 | 0.9533 |
| TN subtype (%) | 7 | 0.0024 | -0.0093–0.0140 | 0.6903 |
| pCR rate (%) | 8 | 0.0099 | -0.0220–0.0418 | 0.5442 |

ER = estrogen-receptor; HER2 = human epidermal growth factor receptor 2; NR = not reported; pCR = pathological complete response; PR = progesterone-receptor; TN = triple-negative
